# Four-dimensional flow MRI for identifying high-risk esophageal varices: a comparison and combination with spleen volume and splenic extracellular volume fraction

**DOI:** 10.1186/s13244-026-02341-1

**Published:** 2026-06-22

**Authors:** Xiaohuan Li, Yuhan Zhang, Zhiyuan Chen, Ke Wei, Yan Zhang, Xiying Zhao, Shuping Zhang, Ruhang Huang, Yupin Liu, Dongjing Zhou

**Affiliations:** 1 The Second Clinical Medical College of Guangzhou University of Traditional Chinese Medicine, Guangzhou, China; 2 Department of Radiology, The Second Affiliated Hospital of Guangzhou University of Chinese Medicine, Guangzhou, China; 3 Integrated Department, The Second Affiliated Hospital of Guangzhou University of Chinese Medicine, Guangzhou, China; 4 Department of Gastroenterology, The Second Affiliated Hospital of Guangzhou University of Chinese Medicine, Guangzhou, China

**Keywords:** Liver cirrhosis, Esophageal variceal, Four-dimensional flow MRI, Spleen volume, Extracellular volume fraction

## Abstract

**Objectives:**

To evaluate the diagnostic performance of four-dimensional flow MRI alone and in combination with spleen volume and splenic extracellular volume fraction (ECV) for identifying high-risk esophageal variceal (HRV) in patients with liver cirrhosis.

**Materials and methods:**

A total of 58 cirrhosis patients who underwent four-dimensional flow MRI and endoscopy were prospectively recruited and were divided into HRV group (*n* = 25) and non-HRV (NHRV) group (*n* = 33). The hemodynamic parameters of the portal vein (PV), superior mesenteric vein (SMV), and splenic vein (SV) derived from four-dimensional flow MRI for identifying HRV were analyzed and compared with spleen volume and splenic ECV. Then a combined model for identifying HRV was constructed by using the least absolute shrinkage and selection operator (LASSO) regression and multivariate logistic regression analysis with stepwise forward.

**Results:**

The peak velocity and maximum pressure gradient of the PV, SMV, and SV, as well as the total volume of the SMV, were significantly greater in the HRV group than in the NHRV group. The SV peak velocity had the highest AUROC for identifying HRV and was comparable to the spleen volume and splenic ECV. The combined model incorporating these three indicators showed superior performance with an AUC of 0.945, outperforming individual imaging indicators and other laboratory-based models.

**Conclusion:**

Four-dimensional flow MRI is a valuable noninvasive technique for detecting HRV in cirrhosis patients. The combined model incorporating SV peak velocity, spleen volume, and splenic ECV provided an accurate prediction of HRV, which may help avoid unnecessary screening endoscopy.

**Critical relevance statement:**

The combined model incorporating spleen vein peak velocity derived by 4D flow MRI, spleen volume, and spleen ECV performed better than individual imaging indicators and laboratory-based models, which may help to avoid unnecessary screening endoscopy and aid in clinical decision-making.

**Key Points:**

- Noninvasive assessment of the risk of HRV in cirrhosis remains crucial but inadequate.
- Four-dimensional flow MRI can effectively identify HRV in patients with cirrhosis.
- Combining SV peak velocity, splenic volume, and ECV effectively identifies HRV.

**Graphical Abstract:**

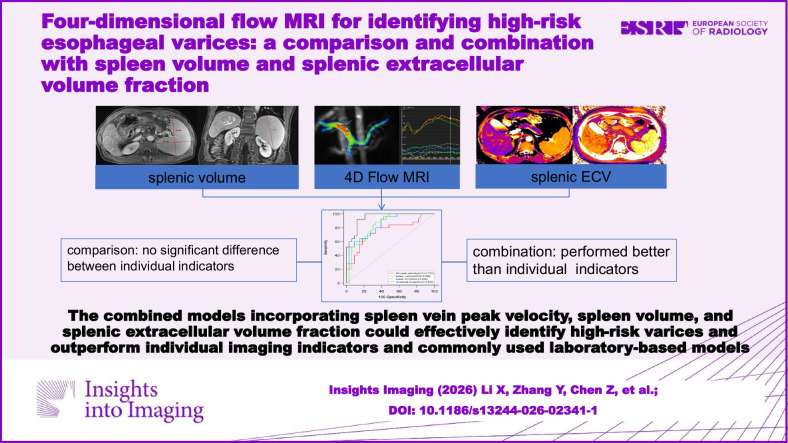

## Introduction

Esophageal variceal (EV) bleeding is a frequent and potentially life-threatening complication in patients with liver cirrhosis, occurring in 10–15% of cases each year [1], with a case fatality rate of up to 20% within six weeks [2]. Early identification of high-risk varices (HRV) is essential for cirrhosis patients, as certain preventative measures, such as nonselective beta-blockers and variceal band ligation, can help prevent EV bleeding and eventually improve overall survival [3, 4]. While endoscopy is the gold standard for risk stratification of EVs, it is invasive and costly and may not be well tolerated by patients for repeated examinations [5, 6]. Therefore, it is imperative to explore noninvasive diagnostic methods for the risk stratification of EVs.

According to the American Association for the Study of Liver Diseases (AASLD) Practice Guideline [7], MR or ultrasound elastography are recommended noninvasive tools for assessing HRV risk. However, the availability and high expense of the device make it difficult to be widely used, especially in economically disadvantaged areas. MRI is commonly used in cirrhosis patients to monitor disease progression and rule out hepatocellular carcinoma, and its role in the risk assessment of HRV has also been explored in recent years. The spleen volume and splenic extracellular volume fraction (ECV) measured on MRI have been shown to be an effective indicator for EV risk assessment in cirrhosis patients [8–12].

Four-dimensional (4D) flow MRI is a novel noninvasive time-resolved phase contrast MR imaging with three-dimensional anatomic coverage and velocity encoding (VENC) along all three flow directions, enabling accurate visualization and quantification of the portal vein (PV), superior mesenteric vein (SMV), and splenic vein (SV) by providing relevant anatomic and hemodynamic information [13]. While some pilot studies have investigated the use of 4D flow MRI for EV assessment and treatment evaluation in cirrhosis patients [14**–**18], its efficacy has not been thoroughly studied, especially compared to other indicators such as spleen volume and splenic ECV.

Therefore, this study aimed to evaluate the effectiveness of 4D flow MRI parameters alone and in combination with spleen volume and splenic ECV for identifying HRV in patients with liver cirrhosis.

## Materials and methods

### Patients

This prospective study complied with the ethical guidelines of the 1975 Declaration of Helsinki and was approved by the ethics committee of our institute (No.YF2022-171-01). Written consent was obtained from all participants. Patients who met the inclusion criteria were consecutively recruited from May 2022 to January 2023. The inclusion criteria were as follows: (1) age ≥ 18 years; (2) a diagnosis of cirrhosis confirmed by hepatologists according to the cirrhosis diagnostic guidelines set by the Chinese Society of Hepatology; (3) scheduled for both liver MRI (for hepatocellular carcinoma surveillance or disease severity assessment) and gastroesophageal endoscopy (for variceal screening per clinical guidelines) within a 2-week interval. The exclusion criteria were as follows: (1) suboptimal MRI quality and incomplete clinical data; (2) patients with transjugular intrahepatic portosystemic shunt, PV thrombosis, cavernous transformation of PV, Budd-Chiari syndrome, acute liver failure, or liver cancer; (3) patients with potential bleeding diseases; (4) previous history of splenectomy; (5) use of gadoxetate disodium as the contrast medium in MR examination.

### MRI protocol

All participants underwent MR examination using a 3.0 T MRI scanner (Magnetom Prisma, Siemens Healthcare) with a standard 32-channel spine array and an 18-channel body array coil. Patients fasted for at least 8 h before MR examination. The full MRI protocol included pre- and post-contrast T1 mapping, dynamic contrast-enhanced T1-weighted imaging, and 4D flow MRI.

The 4D flow sequence utilized prospective ECG-gated and R-wave techniques and covered the vessels of the PV, SV, and SMV. To reduce breathing motion artifacts, adaptive respiratory gating from the signals below was employed. The VENC was adjusted to 30 cm/s for optimal mapping of slow flow in the hypertensive portal circulation. The imaging time for each acquisition was approximately 12 (range: 8–15) min, depending on the respiratory pattern. The 4D Flow sequence was successfully completed in all 58 included patients. Patients with suboptimal image quality due to severe ascites, marked tachycardia (heart rate > 100 bpm), or inadequate respiratory cooperation were excluded prior to enrollment.

T1 mapping was performed before and 10 min after contrast agent injection, covering the entire spleen by using an electrocardiography-gated modified look-locker inversion recovery (MOLLI) sequence. Each patient received 0.1 mL/kg gadobutrol (Gadovist, Bayer Vital GmbH) at a flow rate of 2 mL/s to acquire dynamic contrast-enhanced images. Arterial phase, portal phase, and delay phase images were obtained 15 s, 60–70 s, and 5 min after contrast agent injection, respectively. The acquisition parameters are shown in Supplementary Table S1.

### Image analysis

All imaging data were analyzed by a senior radiologist who was blinded to the clinical and endoscopic data. The maximal width of the spleen, determined as the largest diameter on any transverse section, and the maximal thickness, defined as the largest distance between the inner and outer borders of the spleen perpendicular to the plane of the maximal width, were measured in the cross-section delay phase images. The maximal length, defined as the maximum diameter between the dome of the spleen and the splenic tip, was measured in the coronal plane(shown in Supplementary Fig. S1). Subsequently, the spleen volume was calculated using the following formula [19]: spleen volume (cm^3^) = 30 + 0.58 × (width + length + thickness).

T1 relaxation times of the spleen were measured by drawing six 5–10 mm diameter circular regions of interest (ROIs) on the central three continuous sections (two ROIs per section) in pre- and postcontrast T1 maps, avoiding artifacts, splenic edges, focal lesions, and large vessels(shown in Supplementary Fig. S2). The mean of the six ROI measurements was used as the T1 relaxation time of the spleen. Similarly, T1 relaxation times of the blood pool were measured in the abdominal aorta on the same sections. Then, the splenic ECV was calculated using the following formula:

$${{{\rm{splenic}}}}\; {{{\rm{ECV}}}}=\frac{{1}/{{{\rm{T}}}}{1}_{{spleen}\,{post}}-{1}/{{{\rm{T1}}}}_{{spleen}{pre}}}{{1}/{{{{\rm{T}}}}{1}_{{aorta}\,{post}}-{1}/{{{\rm{T}}}}{1}_{{aorta}\,{pre}}}} \times \left(1-{{{\rm{h}}}}{{\mathrm{ematocrit}}}\right)$$

The 4D-flow data set was postprocessed using flow analysis software (CVI42; Circle Cardiovascular Imaging, Canada). Displacement artifacts and anti-aliasing signals were corrected automatically. The main PV, SMV, and SV were visualized by 3D vector fields and streamlines (shown in Supplementary Video). Three equidistant cuts perpendicular to the vessel’s long axis were made on the vessels of the PV, SMV, and SV, avoiding nonlaminar flow portions (shown in Supplementary Fig. S3). The hemodynamic parameters, including the total volume, peak velocity, and maximum pressure gradient, were measured. The mean of the three measurements of the cut plane was used as the final value.

To evaluate inter-observer consistency, another junior radiologist independently re-measured all MRI assessments. The interobserver reliability of measurements obtained by both radiologists was assessed using the intraclass correlation coefficient.

### Clinical and laboratory data

Demographic and anthropometric data (age, sex) and fasting laboratory indices (platelets, international normalized ratio[INR], activated partial thromboplastin time[APTT], alanine aminotransferase [ALT], aspartate aminotransferase [AST], albumin, total bilirubin [TBIL], direct bilirubin [DBIL], total bile acid [TBA], creatinine [Cr]) were collected within 1 week before endoscopy. The Child–Pugh score, model for end-stage liver disease (MELD) score, AST-to-platelet ratio index (APRI), and fibrosis-4 index (FIB-4) [20, 21] were calculated for each patient (shown in Supplementary Method S1).

### Endoscopy

Esophageal varices were classified independently during endoscopy by a gastroenterologist with more than ten years of experience who was blinded to the clinical and radiological data according to the variceal grading system proposed by Calès [22] as follows: grade 0, no varices; grade 1, varices were flattened by insufflation; grade 2, varices were nonconfluent and protruding into the lumen despite insufflation; and grade 3, confluent varices were not flattened by insufflation. The presence of red signs was also assessed and recorded simultaneously(shown in Supplementary Fig. S4). High-risk esophageal varices were defined as mild esophageal varices (grade 1) with red signs or moderate to severe esophageal varices (grades 2–3) with or without red signs [23]. For the evaluation of inter-observer agreement in endoscopic variceal grading, an independent gastroenterologist re-examined all real-time endoscopies in a blinded manner. The degree of agreement was subsequently analyzed using the weighted kappa statistic.

### Statistical analysis

Continuous variables were reported as the mean ± standard deviation or median (interquartile range [IQR]) and were compared between groups using Student’s *t*-test or the Mann‒Whitney *U*-test, as appropriate. Categorical variables were reported as frequencies (percentages) and were compared between groups using Fisher’s exact test.

Given the limited sample size relative to the number of candidate predictors, variable selection was performed using least absolute shrinkage and selection operator (LASSO) regression, and those variables with *p* < 0.05 were entered into multivariate logistic regression analysis with stepwise forward selection to identify independent predictors. These were then incorporated for the development of a nomogram prediction model. Internal validation was performed using the bootstrap repeated sampling method with 1000 resamples. The optimism-adjusted area under the curve (AUC) was calculated, and a calibration curve was generated following internal verification to assess the consistency. Finally, decision curve analysis was conducted to determine the clinical applicability.

The performance of the different variables for identifying HRV was assessed by measuring the area under the receiver operating characteristic (ROC) curve, sensitivity, and specificity. The Youden index was used to determine the best cutoff value for different variables. For comparison, ROC curves of the variables were plotted, and the corresponding AUC values were compared using the DeLong test.

All the statistical analyses were performed using SPSS (version 26.0; IBM Corp.), R (version 4.3.1; http://www.r-project.org/), and MedCalc (version 20). Statistical significance was defined as a two-sided *p* value < 0.05.

## Results

### Patients

**Fig. 1:**
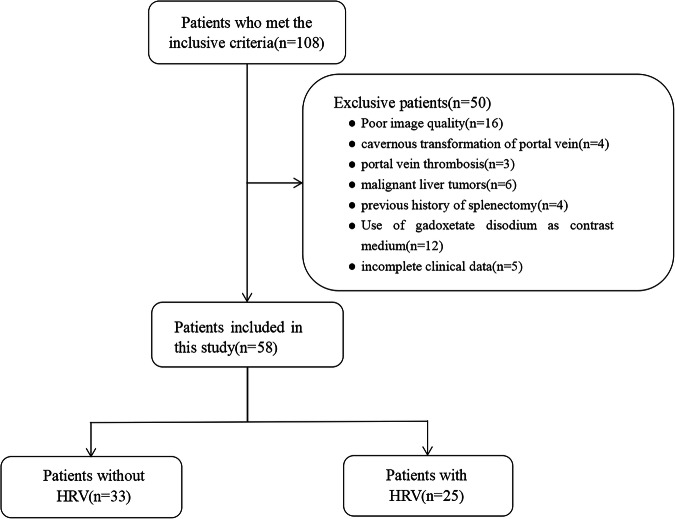
Flowchart of patient inclusion and exclusion. HRV, high-risk varices

Of the 108 consecutive patients with CHB-related cirrhosis who underwent upper abdominal MRI and endoscopy during the study period, 58 were enrolled in this study (Fig. 1). The average age of the participants was 56.58 ± 12.12 years, and 46 (79.3%) were male. According to endoscopy, 25 (43.1%) patients had HRV.

Compared to patients without HRV (NHRV), patients with HRV had significantly greater age, INR, TBIL, DBIL, TBA, Child–Pugh score, MELD score, FIB-4, and APRI, and significantly lower PLT, ALB, ALT, and blood Cr levels. The details of clinical characteristics between the two groups are shown in Table 1.

**Table 1:** Clinical characteristics of cirrhosis patients with and without HRV

Characteristics	NHRV (*n* = 33)	HRV (*n* = 25)	*p* value
Age (year)	52.8 ± 13.1	60.8 ± 9.7	0.013
Sex (%)			0.443
Male	25 (75.8)	21 (84.0)	
Female	8 (24.2)	4 (16.0)	
PLT (10^9^/L)	141 (107–175)	66 (43–97)	< 0.001
INR	1.09 (1.04–1.21)	1.29 (1.19–1.36)	< 0.001
APTT (s)	28.9 (27.9–30.2)	30.4 (28.2–34.1)	0.113
ALT (U/L)	27.0 (18.0–44.5)	19.0 (13.0–29.5)	0.049
AST (U/L)	26.0 (19.0–52.5)	27.0 (21.0–47.0)	0.994
ALB (g/L)	42.9 (37.2–45.0)	36.1 (32.3–40.1)	0.001
TBIL (μmol/L)	14.6 (12.4–24.6)	24.6 (17.1–40.0)	0.010
DBIL (μmol/L)	6.6 (5.1–9.5)	11.1 (8.5–18.2)	0.007
TBA (μmol/L)	9.6 (3.9–29.1)	32.3 (13.4–44.6)	0.002
Cr(μmol/L)	73.0 (59.0–80.5)	70.0 (57.0–93.5)	0.789
Child–Pugh score	6.0 (5.0–6.5)	8.0 (6.0–9.0)	< 0.001
MELD score	4.14 (1.76–6.14)	8.14 (6.06–9.46)	< 0.001
FIB-4	2.02 (1.26–4.15)	5.71 (4.14–9.24)	< 0.001
APRI	0.52 (0.31–1.39)	1.10 (0.78–1.26)	0.010

Fisher's exact probability test was used to compare the difference in categorical variables between the two groups. Student’s *t*-test or the Mann‒Whitney *U*-test, as appropriate, were used to compare the difference in continuous variables among the two groups. Numbers in parentheses represent percentages or IQR

*HRV* high-risk varices, *INR*, international normalized ratio, *NHRV* non-HRV, *PLT* platelets, *APTT* activated partial thromboplastin time, *ALT* alanine aminotransferase, *AST* aspartate aminotransaminase, *ALB* albumin, *TBIL* total bilirubin, *DBIL* direct bilirubin, *TBA* total bile acid, *Cr* blood creatinine, *MELD* model for end-stage liver disease, *FIB-4* fibrosis-4 index, *APRI* AST to platelet ratio index

The inter-observer agreement of endoscopic variceal grading, red sign, and HRV classification by the two gastroenterologists was excellent, with a kappa value range of 0.98 to 1.00. Similarly, there was strong interobserver agreement for the measurements of spleen volume, splenic ECV, and hemodynamic parameters by 4D Flow MRI between the two radiologists (shown in Supplementary Table S2), with intraclass correlation coefficients ranging from 0.661 to 0.998.

### Spleen volume for identifying HRV

**Fig. 2:**
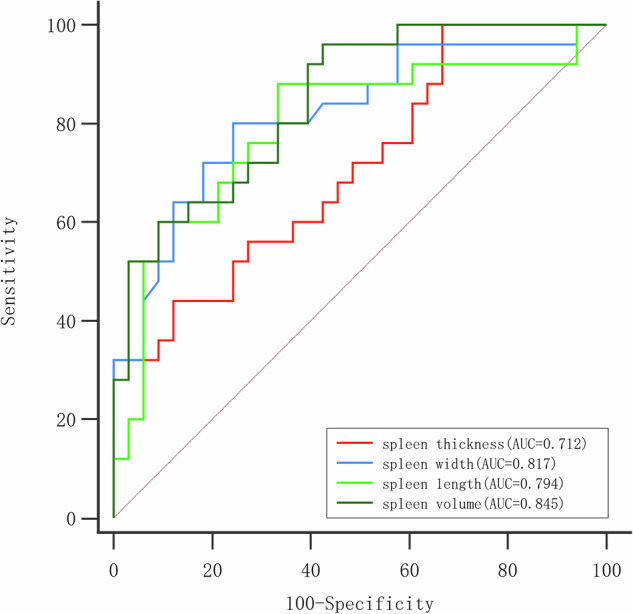
ROC curves of maximal thickness, width, length, and volume of the spleen for identifying HRV. HRV, High-risk varices

As shown in Table 2 and Fig. 2, the maximal width, maximal thickness, maximal length, and volume of the spleen in the HRV group were greater than those in the NHRV group. Of them, spleen volume had the largest AUC of 0.845. Using the cutoff value of 150 cm^3^, the sensitivity and specificity of the spleen volume for identifying HRV were 0.96 and 0.58, respectively.

**Table 2:** The diameter and volume of the spleen in cirrhosis patients with and without HRV

Characteristics	NHRV (*n* = 33)	HRV (*n* = 25)	Cut-off	Sensitivity (95% CI)	Specificity (95% CI)	*p* value
Maximal width (cm)	4.28 ± 0.89	5.83 ± 1.61	4.63	80.0 (59.3–93.2)	75.8 (57.7–88.9)	< 0.001
Maximal thickness (cm)	5.00 ± 1.34	6.23 ± 1.60	3.91	100.0 (86.3–100.0)	33.3 (18.0–51.8)	0.003
Maximal length (cm)	10.00 (9.04–11.14)	11.72 (10.72–14.01)	11.14	72.0 (50.6–87.9)	75.8 (57.7–88.9)	< 0.001
Volume (cm^3^)	147.6 (118.1–217.2)	288.9 (170.6–393.8)	150.0	96.0 (79.6–99.9)	57.6 (39.2–74.5)	< 0.001

*HRV* high-risk varices, *NHRV* non-HRV, *CI* confidential interval

### Spleen T1 mapping and splenic ECV for identifying HRV

Splenic native T1 values were significantly greater in the HRV group than those in the NHRV group. Using the cutoff value of 1312 ms, the sensitivity and specificity of the splenic native T1 value for identifying HRV were 0.88 and 0.58, respectively.

Likewise, the splenic ECV was significantly greater in the HRV group compared to the NHRV group. Using the cutoff value of 35.6%, the sensitivity and specificity of the splenic ECV for identifying HRV were 0.88 and 0.67, respectively.

**Fig. 3:**
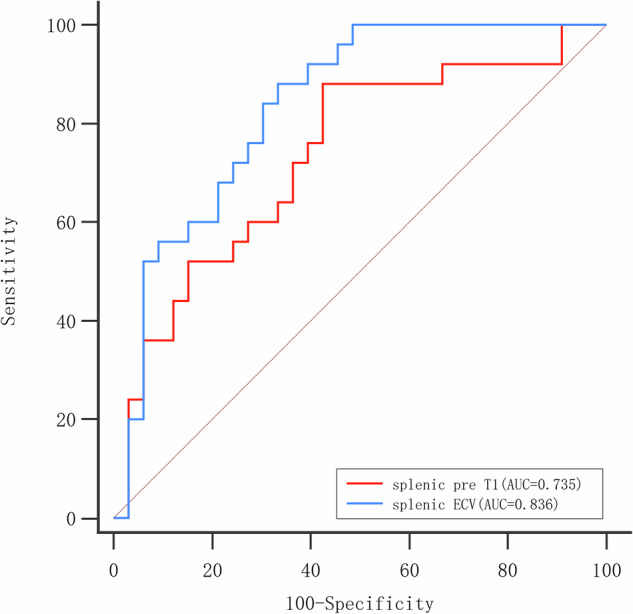
ROC curves of precontrast splenic T1 value and splenic ECV for identifying HRV. No significant difference was shown between these two AUROCs. ECV, extracellular volume fraction; HRV, high-risk varices

Although the splenic ECV had a greater AUC value than the splenic native T1 value, there was no significant difference between the two AUCs according to the DeLong test (*p* = 0.090). In addition, there was no significant difference in the spleen postcontrast T1 value between the two groups. The details are shown in Table 3 and Fig. 3.

**Table 3:** Splenic T1 values and ECV in cirrhosis patients with and without HRV

Characteristics	NHRV (*n* = 33)	HRV (*n* = 25)	Cut-off	Sensitivity (95% CI)	Specificity (95% CI)	*p* value
Precontrast T1 (ms)	1302 (1259–1393)	1463 (1333–1580)	1312	88.0 (68.8–97.5)	57.6 (39.2–74.5)	0.002
Postcontrast T1 (ms)	445 (409–503)	455 (413–494)	377	84.0 (63.9–95.5)	6.1 (0.7–20.2)	0.987
ECV (%)	31.73 (29.70–38.29)	42.46 (36.95–44.19)	35.59	88.0 (68.8–97.5)	66.7 (48.2–82.0)	< 0.001

*ECV* extracellular volume fraction, *HRV* high-risk varices, *NHRV* non-HRV, *CI* confidential interval

### 4D flow MRI for identifying HRV

As shown in Table 4, the peak velocity and maximum pressure gradient of the PV, SMV, and SV were significantly greater in the HRV group than those in the NHRV group. However, only the total volume of the SMV was significantly greater in the HRV group compared to the NHRV group. The total volumes of the PV and SV did not significantly differ between the two groups. ROC analyses revealed that the SV peak velocity had the highest AUC for identifying HRV (shown in Supplementary Table S3).

**Table 4:** Hemodynamic parameters derived from 4D flow MRI in cirrhosis patients with and without HRV

Characteristics	NHRV (*n* = 33)	HRV (*n* = 25)	Cut-off	Sensitivity (95% CI)	Specificity (95% CI)	*p* value
PV						
Total volume (mL)	8.69 (7.08–12.15)	10.03 (8.04–14.42)	7.97	80.0 (59.3–93.2)	39.4 (22.9–57.9)	0.338
Peak velocity (cm/s)	15.49 (13.13–18.61)	18.56 (15.47–24.24)	20.51	48.0 (27.8–68.7)	84.8 (68.1–94.9)	0.015
Maximum pressure gradient (mmHg)	0.10 (0.07–0.14)	0.14 (0.10–0.23)	0.17	48.0 (27.8–68.7)	84.8 (68.1–94.9)	0.016
SMV						
Total volume (mL)	2.15 (1.68–3.17)	3.19 (2.49–6.23)	2.36	80.0 (59.3–93.2)	60.6 (42.1–77.1)	0.010
Peak velocity (cm/s)	11.15 ± 3.09	16.60 ± 6.48	15.6	52.0 (31.3–72.2)	97.0 (84.2–99.9)	< 0.001
Maximum pressure gradient (mmHg)	0.05 (0.03–0.07)	0.11 (0.05–0.19)	0.10	52.0 (31.3–72.2)	97.0 (84.2–99.9)	0.001
SV						
Total volume (mL)	4.58 (3.04–5.74)	5.77 (3.06–11.26)	5.76	52.0 (31.3–72.2)	78.8 (61.1–91.0)	0.162
Peak velocity (cm/s)	12.65 (11.24–16.57)	18.96 (14.92–23.77)	14.14	80.0 (59.3–93.2)	66.7 (48.2–82.0)	0.001
Maximum pressure gradient (mmHg)	0.06 (0.05–0.11)	0.14 (0.09–0.23)	0.08	80.0 (59.3–93.2)	66.7 (48.2–82.0)	0.001

*PV* portal vein, *SMV* superior mesenteric vein, *SV* splenic vein, *HRV* high-risk varices, *NHRV* non-HRV, *CI* confidential interval

The AUROC comparison showed no significant difference between spleen volume, spleen ECV, or SV peak velocity for identifying HRV by using the DeLong test.

### Combined model for identifying HRV

**Fig. 4:**
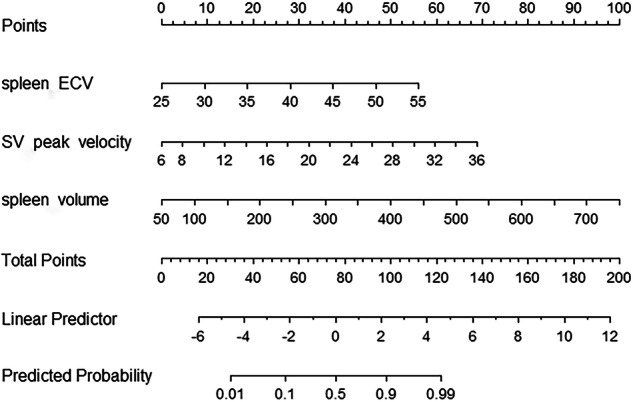
The nomogram to predict HRV in patients with liver cirrhosis. HRV, high-risk varices

All imaging variables for identifying HRV were selected by using LASSO regression. The optimal λ value was obtained by 10-fold cross-validation. Finally, the spleen volume, splenic ECV, SMV maximum pressure gradient, SV peak velocity, and SV maximum pressure gradient were retained. Then, these five variables were subjected to multivariate logistic regression analysis with stepwise forward selection to construct a combined model. Finally, the spleen volume, spleen ECV, and SV peak velocity were input into R software to create a nomogram for predicting HRV (Fig. 4). In this nomogram, a higher score—calculated from the sum of designated scores for each predictor—indicates an increased likelihood of HRV. The calibration curve (Fig. S5) of the nomogram showed a strong agreement in predicting HRV. The apparent C-index for the prediction model was 0.945. Internal validation using 1000 bootstrap resamples yielded an optimism-corrected C-index of 0.940 (95% CI: 0.870–0.989), demonstrating minimal overfitting and good model stability. Additionally, decision curve analysis (Fig. S6) revealed a significant net benefit across nearly all threshold probabilities.

**Fig. 5:**
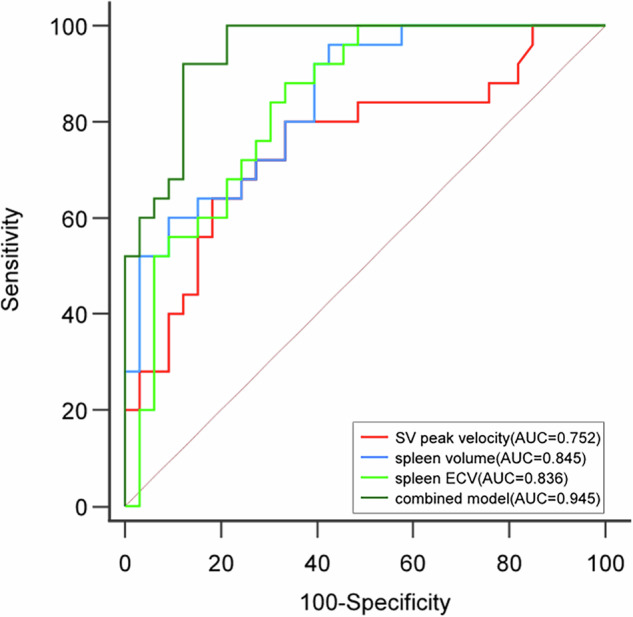
The combined model performed significantly better than the spleen volume, spleen ECV, and SV peak velocity for identifying HRV. ECV, extracellular volume fraction; SV, splenic vein; HRV, high-risk varices

**Fig. 6:**
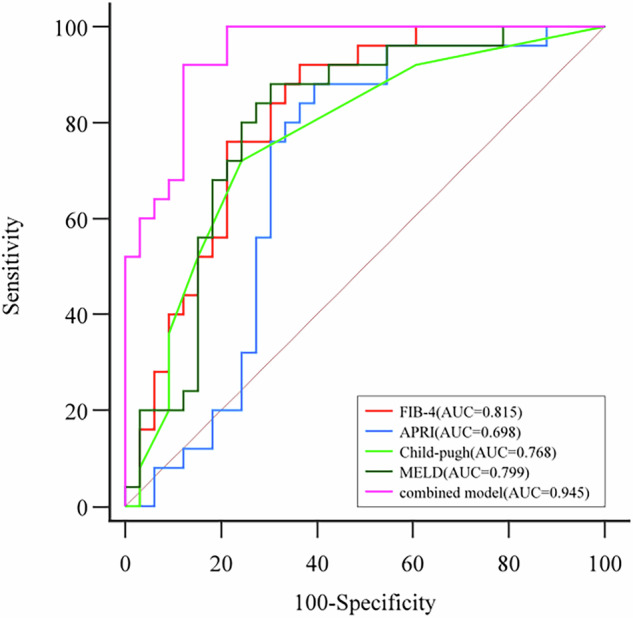
The combined model performed significantly better than the Child–Pugh score, MELD score, APRI, and FIB-4 for identifying HRV. MELD, model for end-stage liver disease; APRI; AST to platelet ratio index; FIB-4, fibrosis-4 index; HRV, high-risk varices

As shown in Fig. 5 and Table 5, the combined model performed significantly better than the spleen volume (*p* = 0.025), spleen ECV (*p* = 0.025), and SV peak velocity (*p* = 0.003) models. Similarly, the combined model outperformed commonly used models based on laboratory tests, such as the Child–Pugh score, MELD score, FIB-4 score, and APRI (shown in Fig. 6).

**Table 5:** The *p* value of DeLong test for comparison of different models to identify HRV

Models	SV peak velocity	Spleen volume	Spleen ECV	Combined model
SV peak velocity	-	0.295	0.355	0.003
Spleen volume	0.295	-	0.895	0.025
Spleen ECV	0.355	0.895	-	0.025
Combined model	0.003	0.025	0.025	-

*HRV* high-risk varices, *SV* splenic vein, *ECV* extracellular volume fraction

## Discussion

In this study, we demonstrated that the hemodynamic parameters derived by 4D flow MRI can effectively predict HRV in cirrhosis patients. Notably, the SV peak velocity exhibited the best diagnostic performance, comparable to spleen volume and splenic ECV. Additionally, 4D flow MRI can serve as a complementary method to other splenic indicators for improved diagnostic efficiency. The combined model incorporating the SV peak velocity, spleen volume, and spleen ECV outperformed individual imaging indicators and established laboratory-based models. These results will provide a basis for clinical decision-making.

While 4D flow MRI is increasingly utilized in the head [24], aorta [25], and heart [26], its application in the PV system is challenged by small vessel calibers, slow flow velocities, and breathing motion [27], with limited data on EV assessment. Motosugi et al [16] reported 100% sensitivity and specificity for HRV using azygous flow > 0.1 L/min plus PV fractional flow change < 0, though their cohort included only 23 patients. In this study, we did not analyze azygous hemodynamics due to suboptimal visualization from a low VENC setting, which may lead to imprecise measurements. Our findings align with those of Karam et al [17] and Hu et al [18], who validated 4D flow MRI for HRV stratification, highlighting the strong diagnostic efficacy of SV peak velocity. Its superior performance over PV hemodynamic parameters may be attributed to its hemodynamic mechanism. Under conditions of elevated PV pressure, multiple collateral vessels develop other than the left gastric vein, leading to substantial fluctuations in PV blood flow due to extensive shunting, whereas SV hemodynamics are more reliable and closely correlated with the left gastric vein, which is widely recognized to play a critical regulatory role in the formation and progression of EV [28]. Additionally, only the SMV volume was notably greater in the HRV group, with no significant between-group difference in PV or SV volume. This could be due to the SMV collecting blood flow from the intestines and mesenteric vessels, which dilate in response to vasoactive substances and increase cardiac output [29]. However, PV and SV volumes were influenced by the highly variable number and size of the spontaneous portosystemic shunts, which are more prevalent in HRV patients.

Portal hypertension increases splenic venous outflow resistance, causing splenic congestion and splenomegaly [30], with subsequent blood pooling, pulp hyperplasia, and fibrosis expanding the extracellular space, which is reflected in increased splenic ECV [31]. Consistent with previous studies [7–11], we found significantly higher spleen volume and splenic ECV in the HRV group. While spleen volume alone showed good diagnostic performance, the combined model provided superior accuracy, indicating that 4D Flow MRI captures functional abnormalities of the PV system that are not fully reflected by splenic enlargement, thus improving HRV risk stratification. Despite the lengthy acquisition time and complex postprocessing of 4D flow MRI, the combined model delivers higher diagnostic accuracy than splenic volume alone. Given the life-threatening consequences of missed HRV, including variceal hemorrhage and mortality, this incremental diagnostic benefit is clinically significant, particularly for specific patient subgroups such as individuals with indeterminate elastography findings. However, further investigations into accelerated techniques to reduce scan time and simplified postprocessing workflows are needed to improve the clinical applicability of 4D flow MRI.

The Baveno VII criteria (B7C) [32] recommends vibration-controlled transient elastography (VCTE)-derived liver stiffness measurement (LSM) for risk stratification of HRV in cirrhosis patients. However, factors like narrow intercostal spaces, massive ascites, liver inflammation and cholestasis can compromise LSM accuracy [33, 34], with a reported 15% technical failure rate [35]. Furthermore, the B7C endoscopy-sparing threshold (LSM < 20 kPa plus platelet count > 150 × 10⁹/L) is stringent, demonstrating a high sensitivity of 97% but a low specificity of 41% [36], leading to excessive unnecessary endoscopies. In contrast, our model, despite a slightly lower sensitivity of 92%, achieved a markedly improved specificity of 88%. However, the lack of LSM data in most patients limited direct head-to-head comparison with the B7C. Further investigations are necessary to evaluate the potential of the model as a second-line tool for patients with indeterminate elastography findings. Additionally, our model may serve as a valuable alternative for patients for whom VCTE is technically unfeasible, as well as in resource-limited settings and non-hepatology centers where VCTE is not routinely available. Nevertheless, it should be emphasized that this study is a single-center investigation with a relatively small sample size. Although bootstrap internal validation was performed, the study remains substantially underpowered. Besides, CHB predominance and use of a 3.0 T Siemens scanner may limit extrapolation to other etiologies, vendors, and field strengths. Accordingly, extreme caution should be warranted prior to clinical implementation before it is validated in large-scale, multicenter studies.

This study had several limitations. First, as the gold standard for portal pressure measurement, hepatic venous pressure gradient was not available in this study. Therefore, the correlation between our predictive model and this metric requires further investigation. Second, the VENC setting in this study was relatively low, limiting the ability to evaluate high-flow vessels such as the hepatic artery and azygous vein. Third, we used the widely accepted Chinese three-category EV grading system rather than the Baveno/AASLD two-tier classification, owing to its long-term use at our center and excellent inter-observer agreement. While prior studies showed no significant differences in diagnostic consistency and HRV detection between the two systems [37], further validation of 4D flow MRI for HRV using Baveno/AASLD criteria is needed. Finally, despite accelerated methods being employed, 4D Flow MRI adds 8–15 min to standard protocols, which must be weighed against its advantages. For patients undergoing MRI for hepatocellular carcinoma surveillance—a common scenario in cirrhotic populations—the additional sequences add marginal cost and burden. However, in settings where MRI is performed solely for HRV assessment, simpler and less expensive alternatives (ultrasound elastography, serum biomarkers) may be more appropriate first-line tools. Cost-effectiveness analysis comparing different screening strategies is warranted in future studies.

In conclusion, 4D flow MRI is a valuable noninvasive technique for detecting HRV in cirrhosis patients. The combined models incorporating SV peak velocity, spleen volume, and splenic ECV could effectively identify HRV and outperform individual imaging indicators and commonly used laboratory-based models, which may help avoid unnecessary screening endoscopy and aid in clinical decision-making for patients with liver cirrhosis.

## Supplementary information

ELECTRONIC SUPPLEMENTARY MATERIAL

Supplementary video

## Abbreviations

AASLD: American association for the study of liver diseases
ALT: Alanine transaminase
APRI: AST to platelet ratio index
AST: Aspartate aminotransferase
B7C: Baveno VII criterion
CI: Confidence interval
ECV: Extracellular volume fraction
EV: Esophageal variceal
FIB-4: Fibrosis-4 index
HRV: High-risk varices
LSM: Liver stiffness measurement
MELD: Model for end-stage liver disease
PV: Portal vein
ROI: Region of interest
SMV: Superior mesenteric vein
SV: Splenic vein
TBIL: Total bilirubin
VCTE: Vibration-controlled transient elastography
VENC: Velocity encoding

## Declarations

### Study design

This study was designed as a prospective study. The aim of this study was to evaluate the application of four-dimensional flow MRI in identifying high-risk EV for patients with liver cirrhosis. Data collection, participant enrollment, and examination procedures were conducted in accordance with a predefined protocol to ensure consistency and minimize bias.
