# Supplementary material for: Four-dimensional flow MRI for identifying high-risk esophageal varices: a comparison and combination with spleen volume and splenic extracellular volume fraction
Source: Insights Imaging. 2026 Jun 22;17:169. doi: 10.1186/s13244-026-02341-1 (PMC13287174; doi:10.1186/s13244-026-02341-1)
Supplement: Supplementary file 1 — ELECTRONIC SUPPLEMENTARY MATERIAL [file 13244_2026_2341_MOESM1_ESM.pdf]

Four-dimensional flow MRI for identifying high-risk esophageal varices: a comparison and combination with spleen volume and splenic extracellular volume fraction

ELECTRONIC SUPPLEMENTARY MATERIAL

Supplementary Table S1. Acquisition parameters of MRI

Sequence	TR (ms)	TE (ms)	Voxel size (mm)	FOV read (mm^3)	Thickness (mm)	Flip angel (degree)
Axial TIWI (pre-contrast)	3.97	1.29/2.52	1.2×1.2×3.0	380	3.0	9.0
Axial TIWI (post-contrast)	3.97	1.23/2.46	0.7×0.7×2.0	380	2.0	9.0
Coronal T1WI (post-contrast)	4.28	1.35/2.58	1.4×1.4×2.0	450	2.0	10.5
T1 mapping (pre-contrast)	279.12	1.12	1.4×1.4×6.0	360	6.0	35
T1 mapping (post-contrast)	359.12	1.12	1.4×1.4×6.0	360	6.0	35
4D Flow MRI	50.64	4.22	3.1×3.1×2.5	400	2.5	7

Abbreviation: TR, repettition time; TE, echo time; FOV, field of view

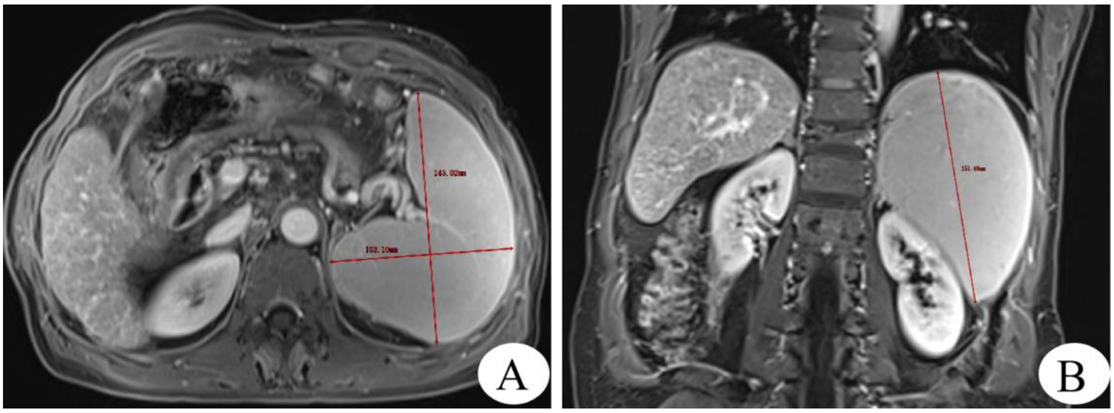

Figure S1. Diagram of measurements of maximal width and maximal thickness of spleen on axial delay T1 weighted image (Figure A) and maximal length of spleen on coronal delay T1 weighted image(Figure B).

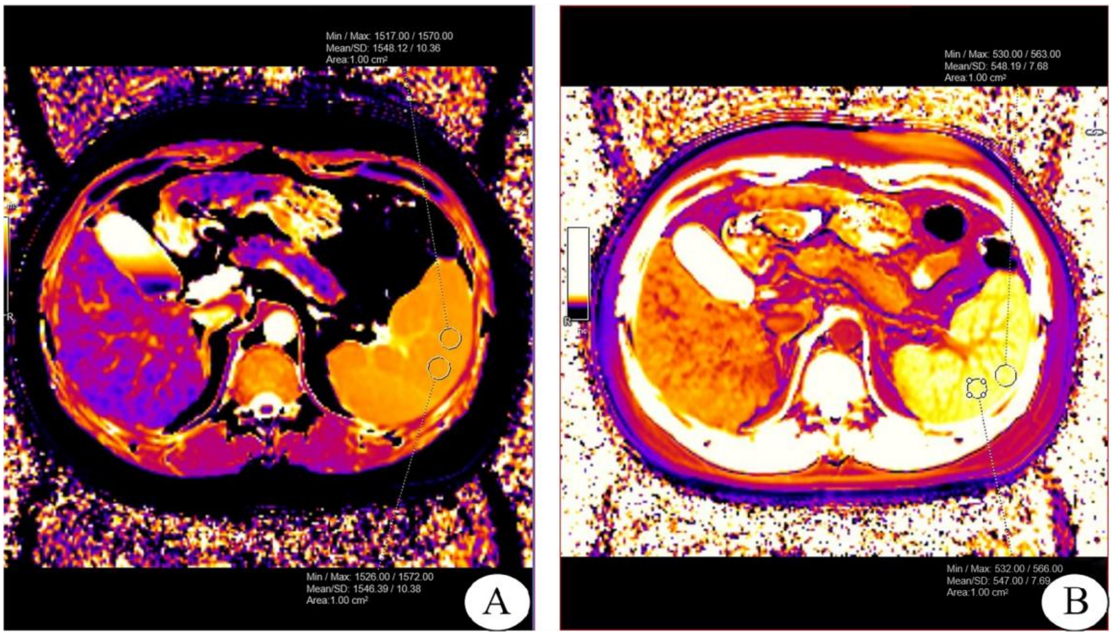

Figure S2. Diagram of ROIs drawn on pre-contrast(Figure A) and post-contrast(Figure B) T1 maps.

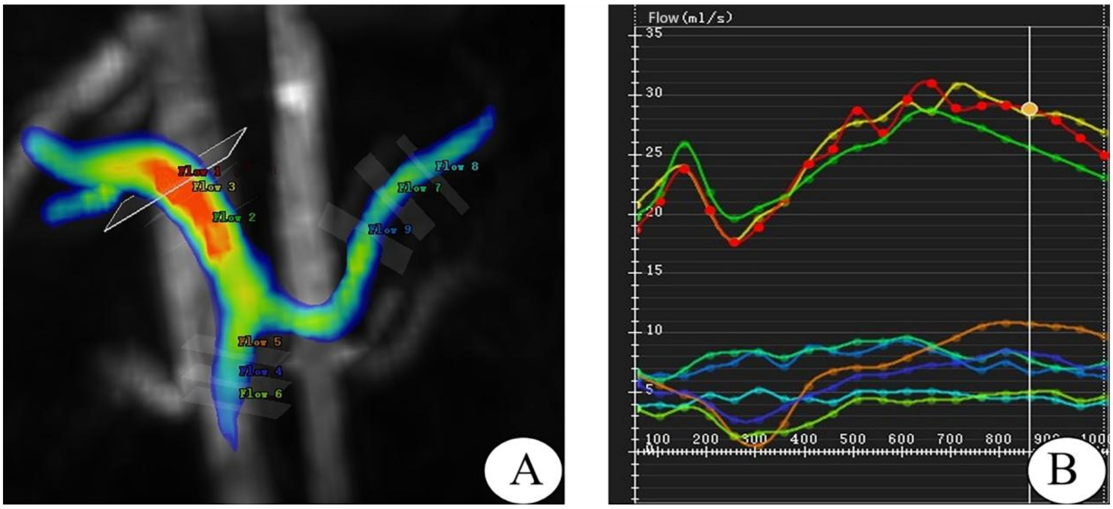

Figure S3 Diagram of three equidistant cuts perpendicular to the vessel's long axis were made on the vessels of the PV, SMV, and SV, respectively(Figure A), the flow analysis software then calculates the hemodynamic parameters automatically(Figure B).

Supplementary Method S1. The calculation of Child-Pugh score, MELD score, APRI and FIB-4

Child-Pugh score：

Index	score		
	1	2	3
hepatic encephalopathy	No	stage1-2	stage3-4
ascites	No	mild	moderate-severe
bilirubin（μmol/L）	＜34.2	34.2-51.3	＞51.3
albumin（g/L）	≥35	28-34	＜28
prothrombin time（s）	≤14	15-17	≥18

MELD=3.8×ln(bilirubin[mg/dl])+11.2×ln(INR)+9.6×ln(creatinine [mg/dl])+6.4×(etiology: 0 if cholestatic or alcoholic, 1 otherwise)

$$\mathrm{APRI} = \mathrm{AST[/upper\ limit\ of\ normal]/platelets\ [10^9/L]} \times 100$$

$$\mathrm{FIB\text{-}4} = \mathrm{age[years]} \times \mathrm{AST[U/L]/(platelets[10^9/L]} \times \sqrt{\mathrm{ALT[U/L]}})$$

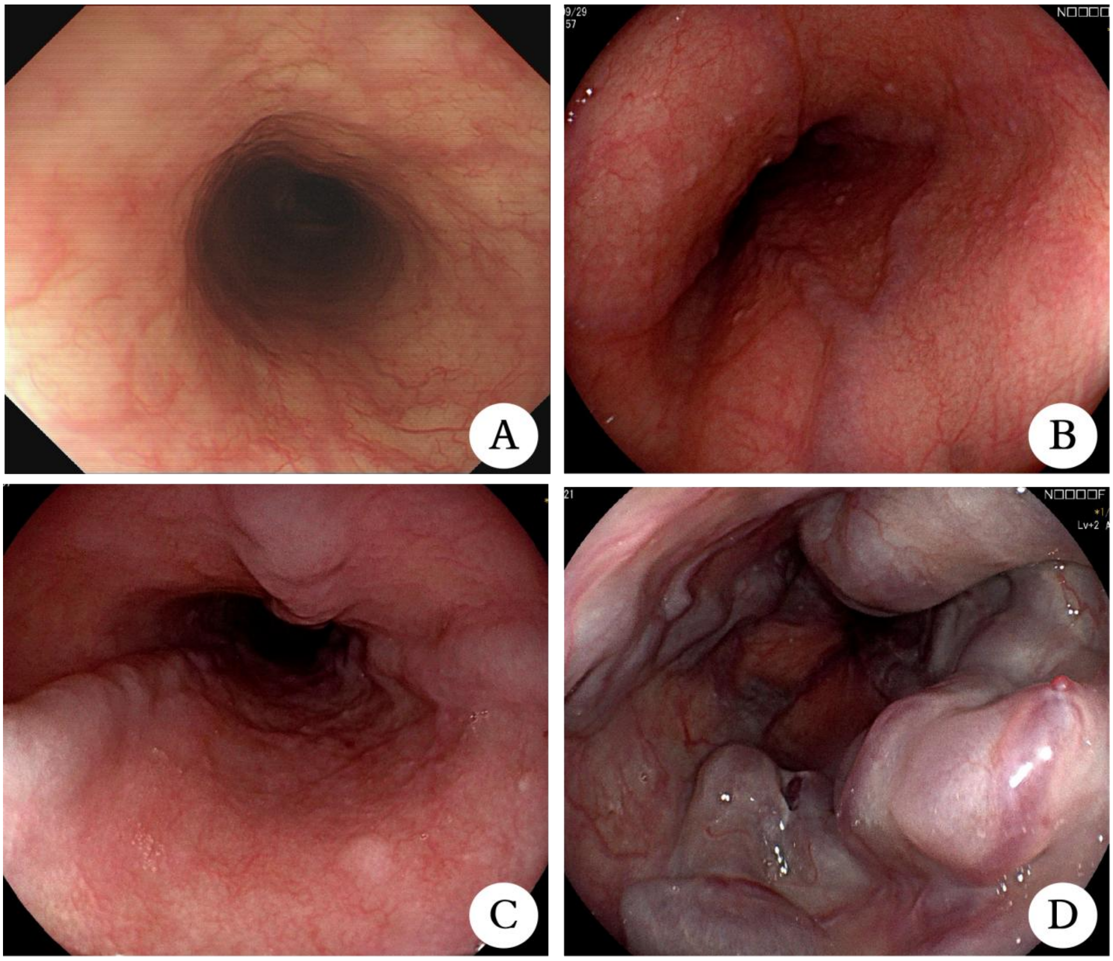

Figure S4 Diagram of classification of esophageal varices . Figure A, B, C and D showed no, mild, moderate and severe esophageal varices in the esophagogastroduodenoscope (EGD), respectively

Supplementary Table S2 . The inter-observer agreement of MR parameter measurements

Characteristics	ICC	95%CI	P value
The diameter and volume of spleen			
maximal width (cm)	0.736	0.591-0.835	<0.001
maximal thickness (cm)	0.661	0.487-0.784	<0.001
maximal length (cm)	0.865	0.783-0.918	<0.001
volume(cm3)	0.959	0.932-0.976	<0.001
Splenic T1 values and ECV			
precontrast T1 (ms)	0.998	0.997-0.999	<0.001
postcontrast T1 (ms)	0.978	0.964-0.987	<0.001
ECV(%)	0.967	0.945-0.980	<0.001
Hemodynamic parameters derived from 4D flow MRI			
PV			
total volume(ml)	0.959	0.932-0.976	<0.001
peak velocity(cm/s)	0.950	0.916-0.970	<0.001
maximum pressure gradient (mmHg)	0.962	0.936--0.977	<0.001
SMV			
total volume(ml)	0.973	0.955-0.984	<0.001
peak velocity(cm/s)	0.864	0.781-0.917	<0.001
maximum pressure gradient (mmHg)	0.850	0.760-0.909	<0.001
SV			
total volume(ml)	0.990	0.983-0.994	<0.001
peak velocity(cm/s)	0.879	0.804-0.927	<0.001
maximum pressure gradient (mmHg)	0.795	0.676-0.874	<0.001

*Abbreviation:*ICC, intraclass correlation coefficient; CI, confidence interval; ECV, extracellular volume fraction; PV, portal vein; SMV, superior mesenteric vein; SV, splenic vein.

Supplementary Table S3. ROC analysis of hemodynamic parameters derived by 4D flow MRI for identifying HRV

Characteristics	AUC	95%CI
PV peak velocity(cm/s)	0.688	0.553-0.803
PV maximum pressure gradient(mmHg)	0.685	0.550-0.801
SMV total volume(L/min)	0.698	0.563-0.811
SMV peak velocity(cm/s)	0.750	0.619-0.854
SMV maximum pressure gradient(mmHg)	0.747	0.616-0.852
SV peak velocity(cm/s)	0.752	0.621-0.856
SV maximum pressure gradient(mmHg)	0.750	0.619-0.855

Abbreviation: HRV, high-risk varices; PV, portal vein; SMV, superior mesenteric vein; SV, splenic vein.

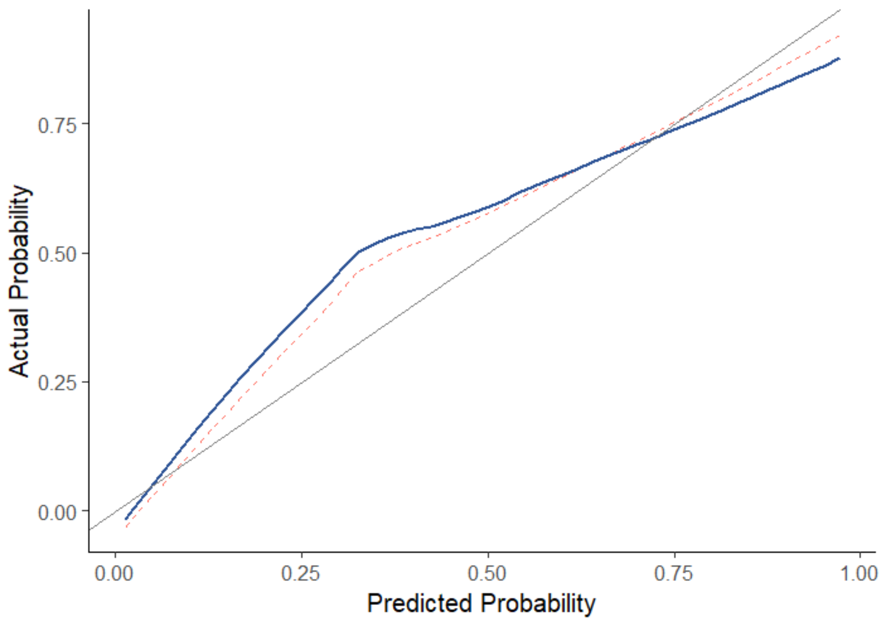

Figure S5 Calibration curve of the prediction nomogram in patients with HRV. Notes: The x-axis represents the nomogram-based predicted probability of HRV, and the y-axis represents the actual probability of HRV. The diagonal dotted line represents an ideal model with no disparities between predicted probability and the actual clinical result. The solid line represents the performance of the nomogram.

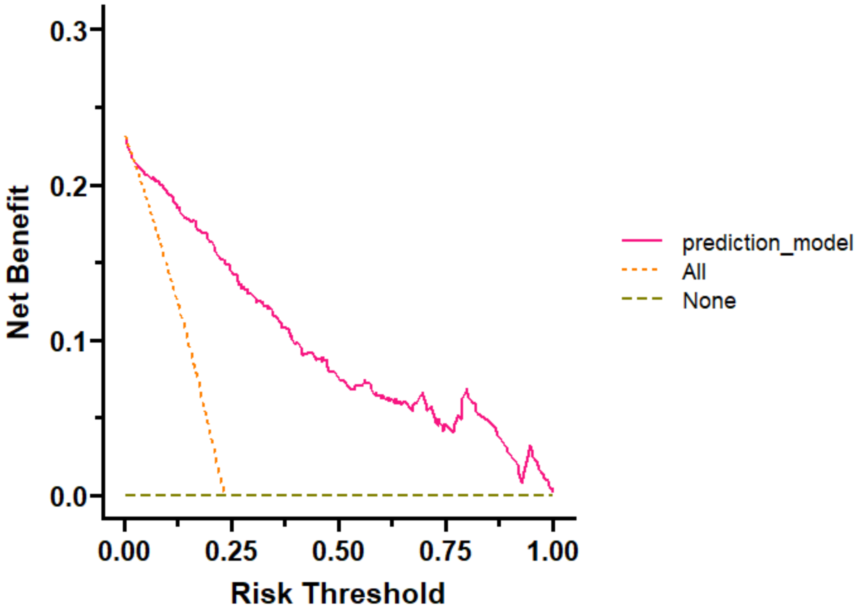

Figure S6 Decision curve analysis of the prediction nomogram. Notes: The x-axis represents the threshold probability, and the y-axis represents the net benefit. The red, orange, and green lines represent the prediction nomogram, the assumption that all the patients will undergo endoscopy, and the assumption that no patients will undergo endoscopy, respectively. The decision curve analysis shows that the nomogram has a good overall net benefit in a wide range of threshold probabilities.
